# New Edge Crush Test Configuration Enhanced with Full-Field Strain Measurements

**DOI:** 10.3390/ma14195768

**Published:** 2021-10-02

**Authors:** Tomasz Garbowski, Anna Knitter-Piątkowska, Aleksander Marek

**Affiliations:** 1 Department of Biosystems Engineering, Poznan University of Life Sciences, Wojska Polskiego 50, 60-627 Poznań, Poland; tomasz.garbowski@up.poznan.pl; 2 Institute of Structural Analysis, Poznan University of Technology, Piotrowo 5, 60-965 Poznań, Poland; 3 Faculty of Engineering and Physical Sciences, University of Southampton, Highfield SO17 1BJ, UK; a.marek@soton.ac.uk

**Keywords:** corrugated cardboard, edge crush test, orthotropic elasticity, digital image correlation, compressive stiffness

## Abstract

The standard edge crush test (ECT) allows the determination of the crushing strength of the corrugated cardboard. Unfortunately, this test cannot be used to estimate the compressive stiffness, which is an equally important parameter. This is because any attempt to determine this parameter using current lab equipment quickly ends in a fiasco. The biggest obstacle is obtaining a reliable measurement of displacements and strains in the corrugated cardboard sample. In this paper, we present a method that not only allows for the reliable identification of the stiffness in the loaded direction of orthotropy in the corrugated board sample, but also the full orthotropic material stiffness matrix. The proposed method uses two samples: (a) traditional, cut crosswise to the wave direction of the corrugated core, and (b) cut at an angle of 45°. Additionally, in both cases, an optical system with digital image correlation (DIC) was used to measure the displacements and strains on the outer surfaces of samples. The use of a non-contact measuring system allowed us to avoid using the measurement of displacements from the crosshead, which is burdened with a large error. Apart from the new experimental configuration, the article also proposes a simple algorithm to quickly characterize all sought stiffness parameters. The obtained results are finally compared with the results obtained in the homogenization procedure of the cross-section of the corrugated board. The results were consistent in both cases.

## 1. Introduction

The increasing consumer demands and absorptive power of the merchant market in today’s world, resulting in the need to pack, store and securely ship more and more various goods, in addition to growing ecological awareness, have led to the increasing interest of manufacturers in cardboard packaging. This fact, in turn, has triggered the inevitable, continuous, and intensive development of numerous corrugated cardboard testing techniques over the last decades.

Assessing the load-bearing capacity of corrugated cardboard products is crucial for their proper design, production final usage, and re-use processes. It is important to emphasize here that corrugated cardboard comprises a few layers, and thus can be called a sandwich structure. Its mechanical properties are directly related to two characteristic in-plane directions of orthotropy, i.e., a machine direction (MD) that is perpendicular to the main axis of the fluting and parallel to the paperboard fiber alignment, and a cross direction (CD), which is parallel to the fluting.

Numerous approaches to sandwich element strength determination, including for corrugated cardboard, can be found in the literature. Analytical methods, starting already in the 1950s, were presented, e.g., in [1,2,3,4,5], whereas numerical methods can be found in [6,7,8,9,10,11], and analytical-numerical techniques in [12,13,14,15,16]. Analytical calculations of the edge crush resistance of cellular paperboard, both in MD and CD, based on the paperboard’s geometric parameters and the mechanical properties of the materials used for its production, was discussed by Kmita-Fudalej et al. [17]. Park et al. [18] investigated the edgewise compression behavior of corrugated paperboard while applying the finite element method (FEM) as well as experimental analysis, i.e., load vs. displacement plots, edge crush tests (ECT) and failure mechanisms. In recent years, methods of artificial intelligence, including artificial neural networks, have become widespread to predict the strength of composite materials, e.g., sandwich structures as presented by Wong et al. [19].

While executing numerical simulations in examining corrugated cardboard, the comprehensive knowledge of each layer’s material properties is necessary. By reason of the anisotropy of the paper-based materials, this is a demanding task. In such a case a good solution is to implement a method called homogenization. This approach efficiently allows us to simplify multi-layer models into single-layered model, described by the effective properties of the composite [9,10,20]. The application of this technique has the benefits of significant savings in computation time while maintaining the accuracy of the results. Hohe [21] presented the strain energy approach as being applicable to sandwich panels for homogenization and proposed an equivalence of a representative element of the heterogeneous and homogenized elements for this purpose.

Another option, in addition to analytical or numerical analysis, for the estimation of corrugated board strength is to carry out measurements from an experiment. Physical testing is very common in the paper industry, and a number of typical tests have been developed to unify the process of the characterization of corrugated cardboard mechanical properties. The aforementioned ECT is used to evaluate the compressive strength, the load during this examination is applied perpendicularly to the axis of the flutes. In the bending test (BNT), four-point bending is executed, two supports are at the bottom of the cardboard whereas two equal forces act on the sample from the opposite side. The shear stiffness test (SST) involves twisting the cardboard cross-section by applying a pair of forces to opposite corners while the other two remain supported. In the torsional stiffness test (TST) the cardboard sample is twisted in both directions. The box compressive test (BCT) is conducted to examine the load bearing capacity of the whole cardboard box [12,13,14,22]. The bursting and humidity tests should also be mentioned here. 

Since ECT is standardized, four different methods have been described, i.e., the edge-clamping method [23], the neck-down method [24], the rectangular test specimen method [24,25,26] and the edge-reinforced method [27,28]. One of the major characteristics which differentiates these tests is the shape of the samples. To assemble the measurements from the outer surfaces of the specimen during the examination, video extensometry can be employed. Such a procedure is based on the measurement of the relative distances between pairs of points traced across images captured at different load values [15]. This is a method comparable to, yet simpler than digital image correlation (DIC) which, as full-field non-contact optical measurement method, is gaining more popularity in the field of experimental mechanics since it ensures very high accuracy of data acquisition. Hägglund et al. applied DIC while examining thickness changes during the ECT of damaged and undamaged panels made of corrugated paperboard [29]. The implementation of DIC for the investigation of the strain and stress fields of paperboard panels subjected to BCT and analysis of their post-buckling behavior was discussed by Viguié et al. in [30,31,32]. A distortional hardening plasticity model for paperboard was presented by Borgqvist et al. [33], who introduced a yield surface characterized by multiple hardening variables attained from simple uniaxial tests. The comparison between the results acquired from the model and the experimental results received while using DIC were demonstrated as well. Combined compression and bending tests of paperboards and laminates for liquid containers while applying DIC were executed by Cocchetti et al. [34,35], who identified the material parameters of anisotropic elastic-plastic material models of foils. For this purpose, inverse analysis was employed while processing the results received from both the experiment and the numerical FEM simulations. DIC and the virtual fields method (VFM) for the recognition of general anisotropy parameters of a filter paper and a paperboard have been discussed by Considine [36]. Åslund et al. applied the detailed FEM for the investigation of the corrugated sandwich panel failure mechanism while performing the ECT and compared the results with the measurements obtained with the use of DIC [37]. Zappa et al. studied the inflation of the paperboard composites which are used in the packaging of beverages while applying DIC [38]. Paperboard boxes with ventilation holes subjected to a compression load were investigated using DIC by Fadiji et al. [39].

It should be pointed out that in a large part of the above-mentioned studies, 3-ply corrugated cardboard specimens were tested. In this study, 5-ply double-wall corrugated cardboard samples were examined. While performing ECT, an optical system with digital image correlation (DIC) is used to determine the displacements on the outer surface of the specimen. The proposed method uses two types of samples, i.e., traditional, cut crosswise to the direction of the wave direction of the corrugated core, and a novel procedure involving a cut at an angle of 45°. Such an approach not only allows for the reliable identification of the stiffness in one direction of orthotropy, but also for the measurement of the full material stiffness matrix, i.e., 4 independent parameters. The obtained results were verified by the results acquired in the homogenization procedure of the cross-section of the corrugated board. As proven, in both cases, the outcomes were very consistent.

## 2. Materials and Methods

### 2.1. Corrugated Cardboard

**Figure 1:**
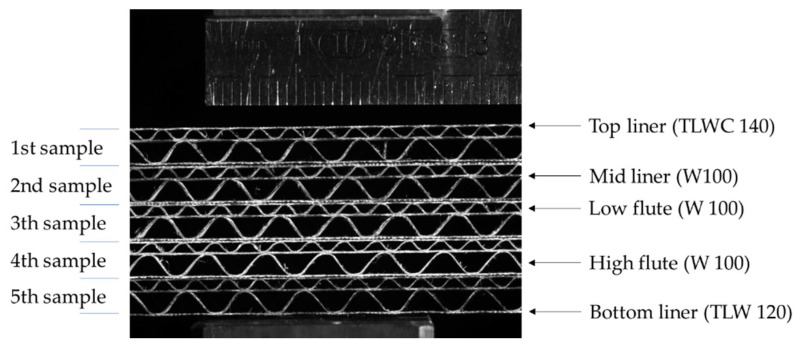
Visualization of 5 samples (stacked on top of each other) of the analyzed corrugated cardboard.

In the current study, a 5-ply corrugated cardboard marked as EB-650 was used. The top liner is made of white, coated, recycled cardboard TLWC with a grammage of 140 g/m^2^. The cross-section has two corrugated layers: (a) low flute (E wave) and (b) high flute (B wave). Both the wavy layers and the flat layer between them, forming the mid liner, are made of lightweight WB cardboard, also recycled, with a grammage of 100 g/m^2^. As a bottom liner, again the white recycled test liner with a grammage of 120 g/m^2^ is used. The geometry of the cross-section of the corrugated board and the configuration of the respective layers are shown in Figure 1, where 5 samples are placed one on top of the other.

Table 1 presents the geometrical parameters of both wavy layers (flutes). The second and third columns of Table 1 shows the wave period (pitch) and the wave amplitude (height), respectively. The take-up ratio, which defines the ratio of the length of the non-fluted corrugated medium to the length of the fluted web, is specified in the last column of Table 1.

Paperboard, which is a main component of corrugated board, is made of cellulose fibers. The orientation of fibers is not random, but rather results from the production process, which causes that their vast majority is arranged along the web, called the machine direction (MD). The second direction, perpendicular to the MD, is called the cross direction (CD). Paperboard is both stronger and stiffer along the fiber direction. 

**Figure 2:**
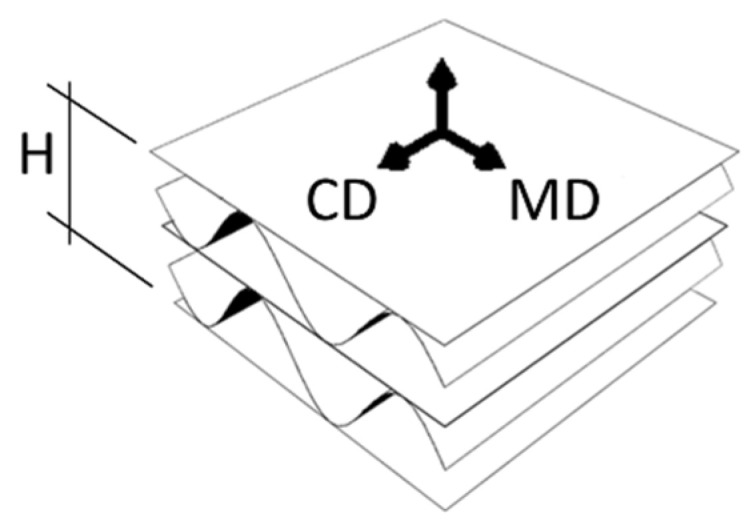
Material orientation in the corrugated board.

In general, materials whose mechanical properties depend on fiber orientation are called orthotropic materials. As a component of corrugated cardboard is paper, it is also able to be considered as an orthotropic material. The orientation of the fibers, shown in Figure 2, makes the corrugated board stronger along the direction of the wave. Thus, the corrugated layers compensate (through the take-up factor) for the weaker mechanical properties of the board in CD.

Table 2 presents the material properties of the individual layers of the corrugated board. The compressive strength in CD, $SCT_{CD}$, is measured while using the short-span compression test according to DIN EN ISO 3037 [26]. The compressive strength of the combined corrugated board in CD, $ECT_{CD}$, specified by the producer–Aquila Września–is 7.6 kN/m (±10%), while the total thickness of the EB-650, H is 4.3 mm (±0.2 mm).

### 2.2. The Edge Crush Test

**Figure 3:**
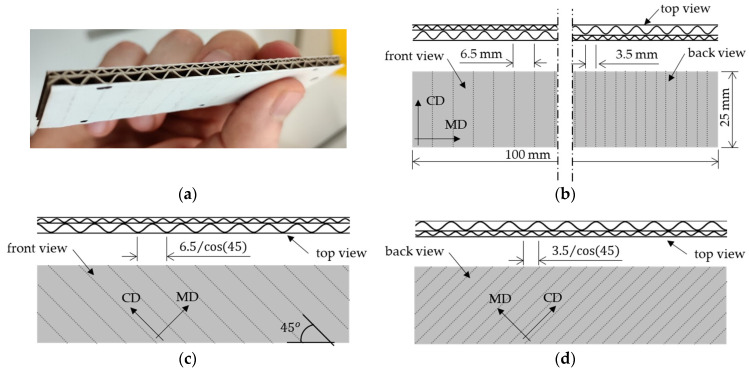
The sample for the standard and new edge crush test: (**a**) standard sample view; (**b**) standard ECT sample–front, back and top view; (**c**) new ECT sample–front and top view; (**d**) new ECT sample–back and top view.

**Figure 4:**
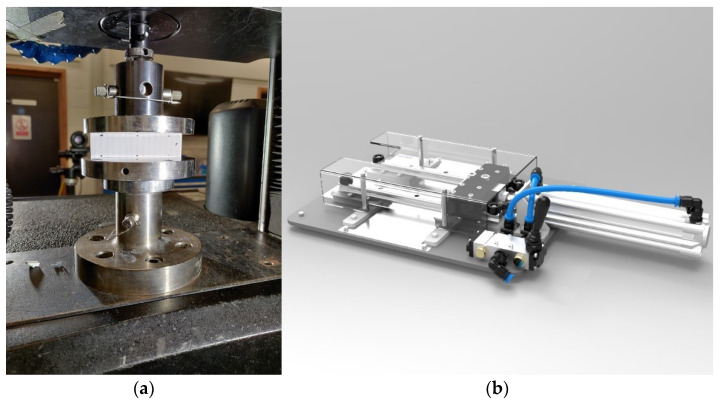
Edge crush test: (**a**) Universal Testing Machine (Instron 5569); (**b**) FEMAT lab device.

The edge crush test (ECT) is a standard test to assess the compressive strength of corrugated board. The test is performed according to FEFCO DIN EN ISO 3037 [25,26], where a 100 mm long and 25 mm high specimen (see Figure 3a,b) is loaded between two rigid plates along its height (see Figure 4a). In order to preserve the parallelism of the cut edges of the sample, it should be cut on a special device, e.g., a FEMat CUT device [22] (see Figure 4b), where the samples are pneumatically cut with one-sided ground blades. All ECT tests were performed under controlled and standard air conditions, i.e., 23 °C and 50% relative humidity.

As already mentioned above, the typical ECT is only used to determine the compressive strength of the corrugated board in CD. Here, the new ECT test setup was also used to determine all of the elastic orthotropic properties of the in-plane tension/compression behavior of corrugated cardboard. For this purpose, beside the traditional method, we also tested samples cut at an angle of 45° to the wave direction (see Figure 3c,d). Since the measurement in standard testing machines is considerably affected by the clearance and susceptibility on the crosshead, non-contact optical techniques are required to credibly measure displacements (deformations or strains).

Additionally, measurement without direct contact does not affect the measurement itself. In contact measurements (e.g., traditional extensometers), noise is introduced into the measurement, which may distort the actual measured values.

### 2.3. Optical Measurements of Sample Deformation

**Figure 5:**
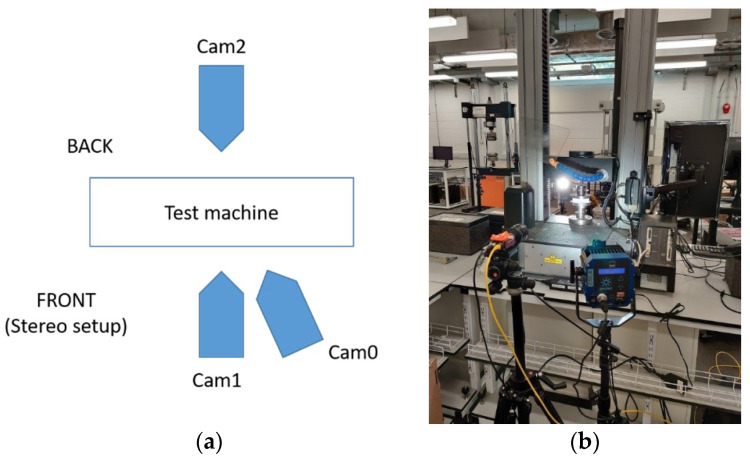
Setup of the optical measurements: (**a**) configuration of cameras on the front and back face; (**b**) cameras recording the front face.

In this study, as mentioned, the specimen was tested while using optical displacement and strain measurements, i.e., virtual extensometry and digital image correlation (DIC). Two cameras (the stereo DIC setup) were employed to track the deformation on the front faces to account for the out-of-plane bending produced by the non-symmetrical section, and single a camera was employed on the back faces for standard optical extensometry, per the test setup shown in Figure 5a. Each of the two faces of the specimen were printed with the speckle pattern for both optical methods, i.e., DIC and video extensometry. Here, three models of deformation measurements were used, namely:

- Crosshead from the machine.
- Stereo (2.5D) DIC on the front (see Figure 5b) plus extensometry on the back.
- Extensometry on the front and back.

The specimen was sandwiched between two platens and aligned using 3D printed L-brackets. Two 5 MPx cameras (Manta G504-b, Allied Vision, Stadtroda, Germany) were used to record greyscale images during the test, see Figure 5. The video extensometry was performed using the MatchID DIC platform (v. 2020.2.0, MatchID, Ghent, Belgium). The cameras were calibrated while applying the MatchID calibration plate (MatchID, Ghent, Belgium) to acquire the pixel (px) to mm conversion rate of ~50 µm/px. The specimen was manually preloaded with a very small load (15 N) to ensure that both edges of the specimen were touching the loading plates. Then, the measured load cell and the displacement were zeroed, and the L-brackets supporting the sample were removed. The load and the crosshead displacement were synchronized with the cameras. The accuracy of the measurement was estimated using a set of 25 static images (without any movement); the standard deviation of the measured elongation was evaluated to be 4 µm, which can be considered the level of uncertainty. The optical displacements were averaged for each face and compared against the crosshead displacement.

In total, 5 samples in CD and 5 samples using the 45° direction were tested. Unfortunately, data from one of the samples in the CD experiment were not recorded properly on the PC and were removed from the statistics. The loading rate was set to 5 mm/min (which is different from the standard rate 12.5 mm/min) because the samples failed too quickly for cameras to get enough data.

**Figure 6:**
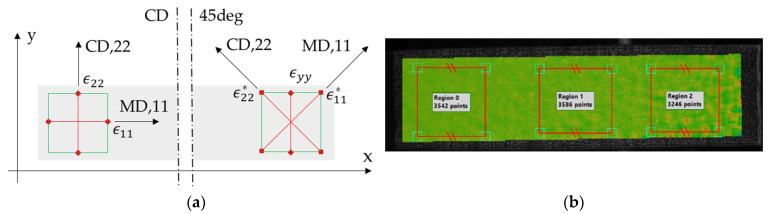
Virtual optical gauges (**a**) sample in CD and in 45°; (**b**) ROI visualization. The * denotes a material orientation in the sample cut in the 45°.

The following stereo DIC procedures, with camera ”Cam1“ as the main, were utilized in this research:

- Perform DIC on the sample’s face while using images from Cam1 and Cam0; region of interest (ROI) visible in Figure 6b.
- Align the data coordinate system with the specimen material direction, i.e., 11=MD, 22=CD, yy= vertical (see Figure 6a).
- Calculate strain from the displacements.
- Select a subregion and extract the data; all data in the subregion is averaged giving one value of desired quantities per image, namely: $\varepsilon_{11},\;\varepsilon_{22},\;\varepsilon_{12},\;\varepsilon_{yy}$.
- Shear strains reported as tensor shear strain component $\varepsilon_{12}$, need to be doubled for the engineering component.

**Figure 7:**
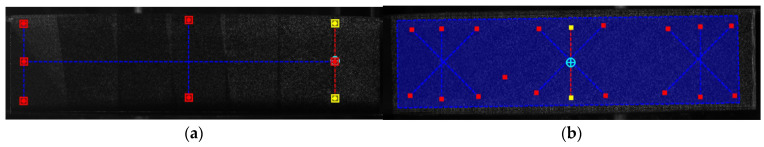
Virtual optical gauges (**a**) sample in CD; (**b**) sample in 45°.

On the other hand, the video extensometry main procedures utilized in this study, were as follows:

- Use a speckle pattern compatible with DIC (pen marks would work equally well, per [15]).
- Only perpendicular cameras were used (front = Cam1, back = Cam2).
- Length of vertical gauges was 350 px (see Figure 6 and Figure 7), while the length of the gauges in the 45° direction were chosen to be 490 px, which is ×1.4 of the vertical gauge (see Figure 6a and Figure 7b).
- The three gauges in their respective directions were averaged to produce a single value of strain, i.e., $\varepsilon_{11}$, $\varepsilon_{22}$ and $\varepsilon_{yy}$ in the 45° direction tests, or $\varepsilon_{11}$ and $\varepsilon_{22}$ in the CD tests.
- All membrane strain is the average of the front and back strains. Ideally, it should be obtained from the trapezoidal distribution of the paperboard cross-section under combined compression/bending. Here, it was simply averaged.
- The shear strain can be calculated from the strain gauge rosette (see Figure 7b): $\varepsilon_{12}=\varepsilon_{yy}-0.5\left(\varepsilon_{11}+\varepsilon_{22}\right)$.

Using the tests for CD, $\varepsilon_{yy}$ (in the CD direction) and $\varepsilon_{xx}$ (in the MD direction) were measured from each image either by averaging large region from the DIC (see Figure 6b) or by using virtual extensometers: 3 vertical plus 1 horizontal (see Figure 7a). The front and back data were averaged to remove artificial bending data. A similar methodology was used in case of the ECT in a 45° direction. All stiffnesses, e.g., $F_{yy}$ vs. $\varepsilon_{yy}$ were calculated from the linear portion of the graphs.

### 2.4. Proposed Method to Identify Matrix ***A***

The identification of matrix **A** is based here on two sets of tests, namely: (a) the standard ECT, in CD and (b) the new ECT in 45° direction. The well-known relation between cross-sectional forces and general strains has the form:

(1) $$\left[\begin{array}{c} \sigma_{11}\\ \sigma_{22}\\ \sigma_{12} \end{array}\right]=\left[\begin{array}{ccc} A_{11} & A_{12} & 0\\ A_{12} & A_{22} & 0\\ 0 & 0 & A_{66} \end{array}\right]\left[\begin{array}{c} \varepsilon_{11}\\ \varepsilon_{22}\\ \varepsilon_{12} \end{array}\right],$$

where $\sigma_{ij}$ are the components of the sectional force vector, in [N/mm]; $A_{ij}$ are the stiffness components, in [N/mm]; and $\varepsilon_{ij}$ are the membrane (in-plane) strains.

From Equation (1) two sets of equations can be extracted, namely in the CD test:

(2) $$\begin{array}{c} A_{12}\varepsilon_{11}+A_{22}\varepsilon_{22}=\sigma_{22},\\ A_{11}\varepsilon_{11}+A_{12}\varepsilon_{22}=0, \end{array}$$

and in the 45° direction test:

(3) $$\begin{array}{c} A_{11}\varepsilon_{11}+A_{12}\varepsilon_{22}=\sigma_{11}^{45}=0.5\sigma^{45},\\ A_{12}\varepsilon_{11}+A_{22}\varepsilon_{22}=\sigma_{22}^{45}=0.5\sigma^{45}. \end{array}$$

By building up a matrix of those equations from two experiments and solving it in the least square sense (se e.g., [40]) the components of matrix $A=\left[A_{11},\;A_{12},\;A_{22}\right]$ can be easily obtained. Component $A_{66}$ can be obtained independently, from the ECT in the 45° direction.

If one uses stresses instead of sectional forces, the following equations can be derived from the test in the CD:

(4) $$\left[\begin{array}{cc} \frac{E_{11}}{1-\nu_{12}\nu_{21}} & \frac{E_{22}\nu_{12}}{1-\nu_{12}\nu_{21}}\\ \frac{E_{11}\nu_{21}}{1-\nu_{12}\nu_{21}} & \frac{E_{22}}{1-\nu_{12}\nu_{21}} \end{array}\right]\left\{\begin{array}{c} \varepsilon_{11}\\ \varepsilon_{22} \end{array}\right\}=\left\{\begin{array}{c} 0\\ \sigma_{22} \end{array}\right\},$$

and from the test in the 45° direction:

(5) $$\left[\begin{array}{cc} \frac{E_{11}}{1-\nu_{12}\nu_{21}} & \frac{E_{22}\nu_{12}}{1-\nu_{12}\nu_{21}}\\ \frac{E_{11}\nu_{21}}{1-\nu_{12}\nu_{21}} & \frac{E_{22}}{1-\nu_{12}\nu_{21}} \end{array}\right]\left\{\begin{array}{c} \varepsilon_{11}^{*}\\ \varepsilon_{22}^{*} \end{array}\right\}=\frac{1}{2}\left\{\begin{array}{c} \sigma_{45}\\ \sigma_{45} \end{array}\right\}.$$

From the test in the CD only, just two constitutive components can be computed, namely Poisson’s ratio:

(6) $$\nu_{21}=-\frac{\varepsilon_{11}}{\varepsilon_{22}},$$

and the elastic modulus:

(7) $$E_{22}=\frac{\sigma_{22}}{\varepsilon_{22}}.$$

On the other hand, from both the CD and 45° tests, all orthotropic stiffness coefficients can be obtained, namely elastic stiffness in MD:

(8) $$E_{11}=-\frac{\sigma_{22}\sigma_{45}}{\varepsilon_{11}\sigma_{45}-2\varepsilon_{11}^{*}\sigma_{22}},$$

elastic stiffness in CD:

(9) $$E_{22}=\frac{\sigma_{22}}{\varepsilon_{22}},$$

Poisson’s ratio $\nu_{12}:$

(10) $$\nu_{12}=\frac{\epsilon_{11}\sigma_{45}}{\varepsilon_{11}\sigma_{45}-2\varepsilon_{11}^{*}\sigma_{22}},$$

Poisson’s ratio $\nu_{21}$:

(11) $$\nu_{21}=1-\frac{2\varepsilon_{22}^{*}\sigma_{22}}{\varepsilon_{22}\sigma_{45}},$$

or using the symmetry principals:

(12) $$\nu_{21}=\nu_{12}\frac{E_{22}}{E_{11}}.$$

The stiffness in the 45° direction can be computed directly from the test in 45° direction:

(13) $$E_{45}=\frac{\sigma_{45}}{\varepsilon_{yy}},$$

and is used to compute the last missing coefficient, namely the in-plane shear stiffness:

(14) $$G_{12}=\left(\frac{2\nu_{12}}{E_{11}}-\frac{1}{E_{11}}-\frac{1}{E_{22}}+\frac{4}{E_{45}}\right).$$

## 3. Results

### 3.1. The ECT Enhanced with Optical Measurement Techniques

**Figure 8:**
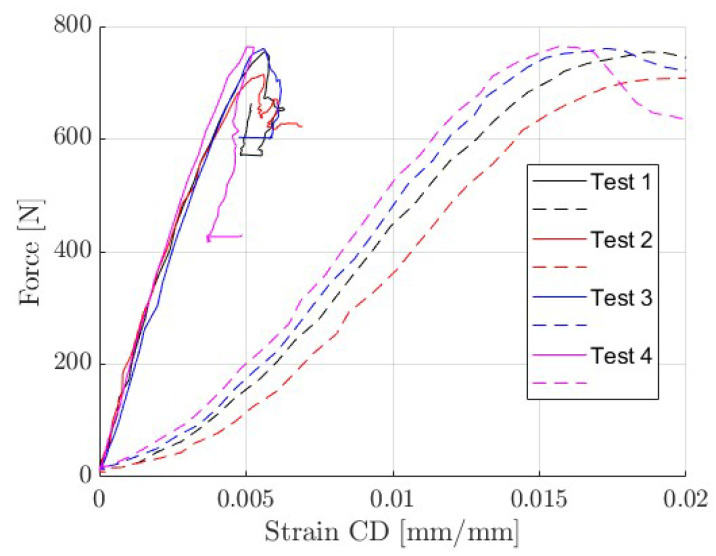
Force-displacement curves. Optical extensometry–solid lines; from machine crosshead–dashed lines.

First, four tests of the CD are presented. Figure 8 shows the differences in the displacements measured by optical techniques (solid line) and taken from the machine crosshead (dashed line).

Table 3 shows the elastic stiffness index, which was computed from the linear part of the curves shown in Figure 8. It should be pointed out that the cross-sectional force is normalized by the sample length (L=100 mm) but not by the sample thickness. This approach complies with the specifications of the corrugated board manufacturers and allows the presentation of results regardless of the sample thickness.

### 3.2. DIC vs. Extensometry

**Figure 9:**
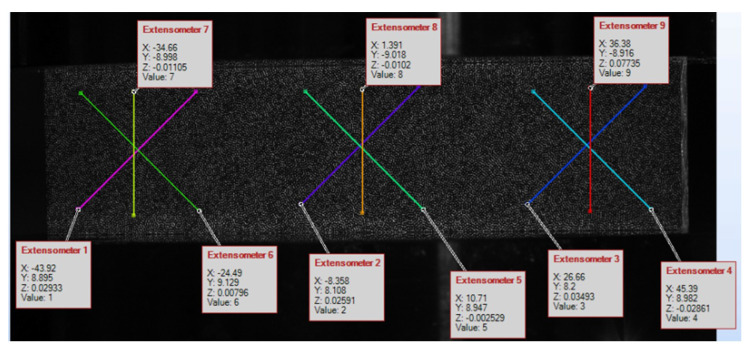
Location of each strain gauge on the sample in the test in the 45° direction.

**Figure 10:**
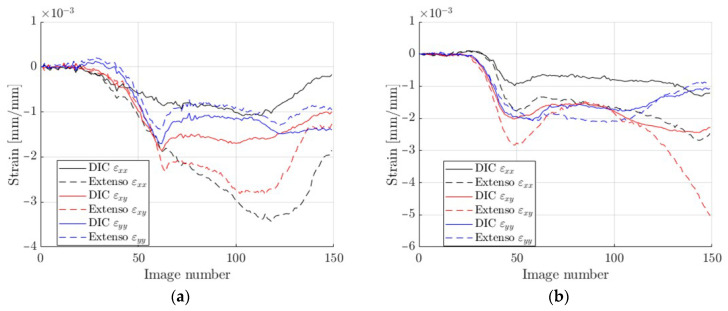

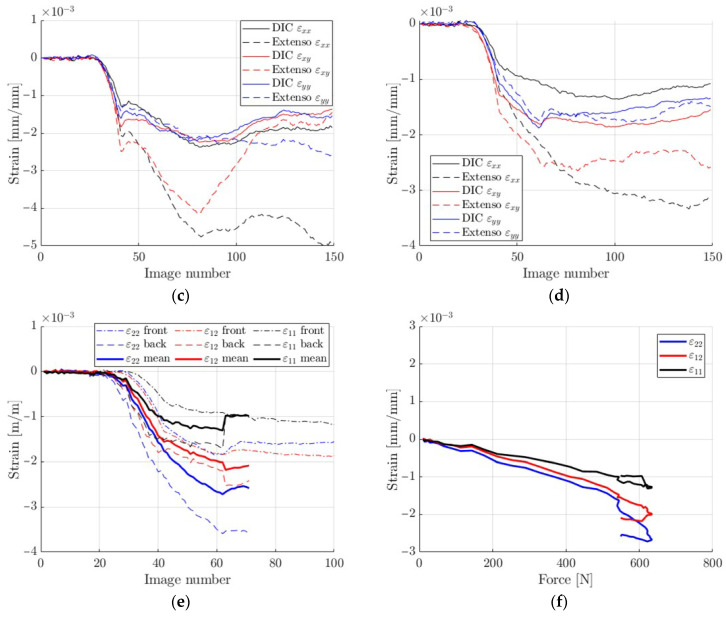
DIC vs. virtual extensometry comparison: (**a**) region 1; (**b**) region 2; (**c**) region 3; (**d**) mean from 3 regions; (**e**) back-to-front average; (**f**) strains resulting from forces.

Then the stereo DIC and the extensometry approach were compared. For this analysis, the selected test in the direction 45° was carefully analyzed. The DIC data in the zones occupied by extensometers were averaged and compared (see Figure 9 and Figure 10).

The results presented in Figure 10 are comparable, but not identical in terms of elasticity, mainly due to a certain inhomogeneity in the deformation caused by the crushing of the edges, which obviously affected the extensometers. However, this can be reduced, e.g., by shortening the gauge length, which appears to be a key a priori choice. The question of how long the extensometers should be is discussed in the next subsection. 

It is known that the error in strain measurements comes from error in the measured displacements (here it is constant at ~0.01 px) and the length of the gauge. Although it seems that the longer the gauge, the better, but the longer the gauge, the greater the risk of taking into account the edge effects of the sample, where (especially in the case of unwaxed samples) the largest local deformations (i.e., crushing and wrinkling) are usually concentrated.

### 3.3. Length of Virtual Extensometry

A study on the length of the optical extensometry was performed on the test number 3 data in the CD–full-field data was extracted (i.e., strains and displacements). Virtual extensometers were generated with varied lengths at different horizontal positions and compared against the averaged vertical strains from the DIC. For example, two points were selected in the center of the sample: one at $Y_{1}=+10$ mm with respect to the center of the sample height, the other at $Y_{2}=-10$ mm and the extensometer strain was calculated from $\varepsilon_{yy}=\left(v_{1}-v_{2}\right)/20$.

**Figure 11:**
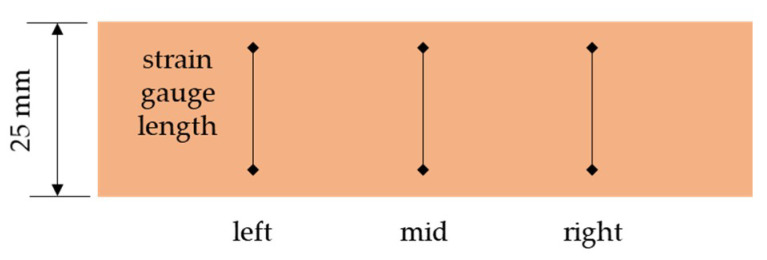
Location of the virtual strain gauges.

Three horizontal positions of the virtual strain gauges were considered: (1) left at 25% of the width; (2) mid at 50% and (3) right at 75% of the sample width. They were also averaged. Figure 11 shows the location of the optical strain gauges. The length of each gauge varies from 4 to 20 mm.

**Figure 12:**
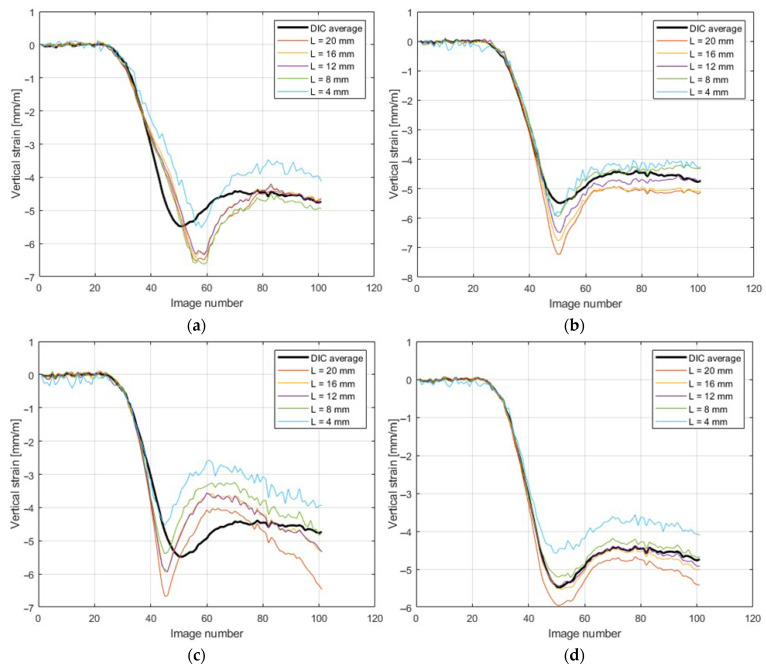
Comparison of strains measured by different lengths of virtual gauges with DIC measurements. (**a**) left set; (**b**) mid set; (**c**) right set; (**d**) averaged.

Figure 12 shows a comparison of strain calculated while using different lengths of virtual gauges with the DIC measurements.

The main observation was that for the test in the 45° direction, the extensometers should be arranged in a rectangular configuration (15 mm×15 mm box, with longer gauges on the diagonal) or circular gauges (so as to keep the gauge length of 15 mm).

### 3.4. Consistency of Tests in 45 Deg Direction

**Figure 13:**
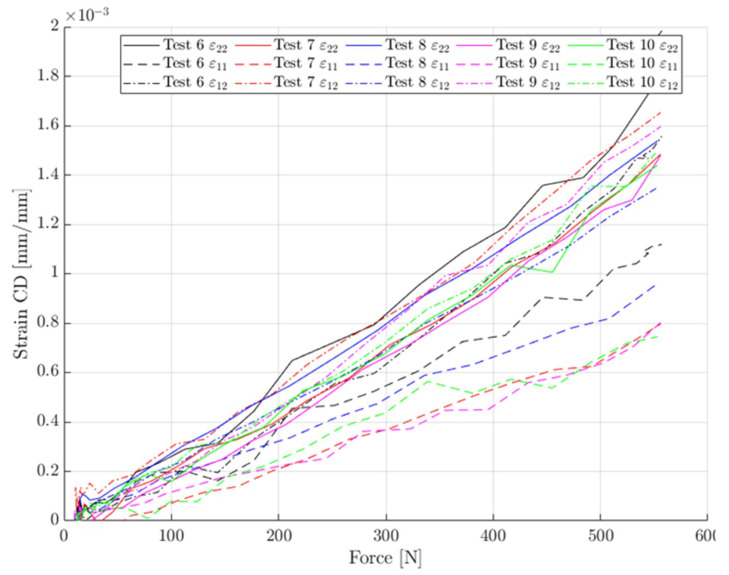
The consistency of the data from tests 6–10.

**Figure 14:**
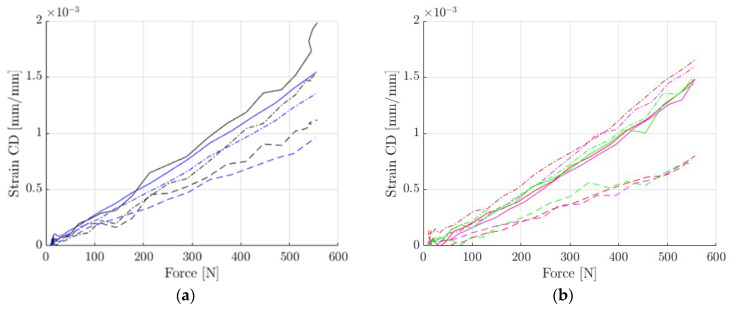
The consistency of data in tests 6–10: (**a**) group 1 (tests 6 and 8); (**b**) group 2 (tests 7, 9 and 10).

The last issue was to check the data consistency of the new test in the 45° direction. For all the CD tests, the force-strain data was very consistent, but unfortunately this was not the case for the 45° tests. For each recorded level of the force, the measured strain components averaged back-to-front are plotted (see Figure 13). It is visible that the tests can be split into two, more consistent groups (see Figure 14). Group 2 had a stiffer response in the 11 (MD) direction.

The reasons for the difference are not fully clear. One of the observations was that group 1 (i.e., test 6 and 8) had a high flute oriented towards the stereo DIC setup (front face as depicted in Figure 3c). Local buckling on that face is more pronounced and that could have affected the measured strain. However, even when using extensometers instead of full DIC, the trend stayed the same. Group 1 had (accidentally) a different orientation of fluting with respect to the plate than group 2 (Figure 3c,d).

### 3.5. Full Matrix A Identification

First, by combining tests 2 and 6 and using Equations (2) and (3) with the least square approximation, one can identify the full A matrix (see Table 4).

The Poisson’s ratio computed directly from the CD test (see Equation (6)) turned out to be ~0.07, which is much closer to the value cited here: $A_{12}/A_{22}=0.09$. In all cases, force was normalized by specimen width (100 mm). In the investigation, test number 1 was removed from the data pool due to an artefact point.

Finally, the same procedure as above was used, but with the two separate groups discussed in previous subsection shown in Figure 14. In total, 178 (group 1) and 204 (group 2) points were used here to calculate the in-plane stiffnesses $\left(A_{11},\;A_{12},\;A_{22}\right)$. This separation made it possible to study the effects of positioning unsymmetric samples on the ECT apparatus.

**Figure 15:**
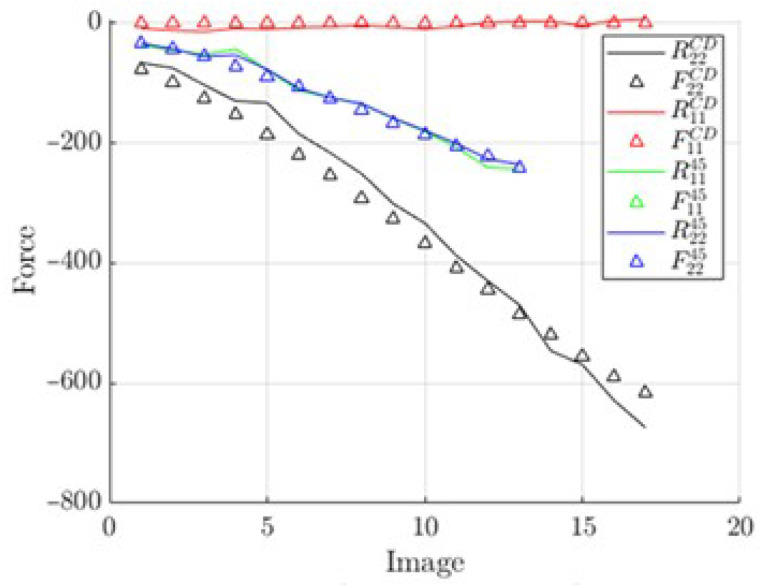
Curves reconstructed from the identified A matrix vs. measured force.

**Figure 16:**
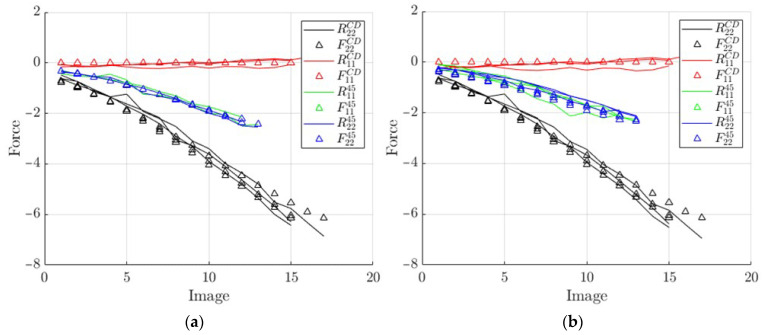
Curves reconstructed from the identified A matrix vs. measured force: (**a**) using tests in group 1; (**b**) using tests in group 2.

The reconstructed elastic forces from the identified parameters are shown in Figure 15 and Figure 16—multiple lines represent multiple tests. These data show good model fitting.

## 4. Discussion

The previous section provides the outcomes of the research, presenting, among others, typical ECT results enriched with digital image correlation and/or optical, virtual extensometry techniques. The results summarized in Table 3 clearly show that the use of the displacements obtained from the machine crosshead introduces an error in the estimation of the stiffness index, underestimating this value almost 3 times. The same observation can also be found in the recent work of Garbowski et al. [15]. The compressive strength given in Table 3 (shown in column 4) is consistent with the value provided by the manufacturer of the corrugated board, namely 7.6 N/mm ±10%.

The comparison of strains obtained from the DIC and while using virtual extensometers is presented in Figure 10. These results were comparable, but not identical. The best fit can be observed for the vertical strain $\varepsilon_{yy}$. Based on the observations regarding the length of the optical extensometer and its influence on the accuracy of the results, 15 mm segments were used for further analyses. This can be observed in Figure 12, where the calculated strains were compared while using DIC and extensometers of different lengths. The main conclusion is that when applying longer gauges, the results are more stable. However, if the optical extensometer is too long (i.e., longer than 15 mm) or too short (i.e., shorter than 8 mm), the differences can be as high as 15%.

**Figure 17:**
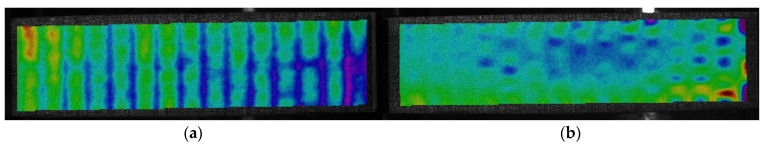
The ECT sample during the CD test: (**a**) sample during the CD test–no buckling; (**b**) sample during the CD test–buckling.

The use of extensometers with a length of ~20 mm causes false results due to the proximity of the measuring tip to the crushed edge of the sample (which is 25 mm high). On the other hand, the use of short gauges of ~5 mm is affected by larger noise and causes the measurements to have an error due to buckling from the plane of the sample (see Figure 17b). The moment when the sample buckles is shown in Figure 12d–image number 38 (for a strain gauge 4 mm long). The influence of buckling (which manifests in the form of an out-of-plane deformation) on the measurement of in-plane deformations can be easily eliminated using the stereo DIC procedure. However, if optical extensometry is to be used, a fairly large area where the results obtained with the extensometer match those obtained with the DIC should be in the range of 8–16 mm.

Table 4 shows the identified components of matrix A. The second column shows the results obtained during tests 2 and 6, while columns 3 and 4 show the results obtained while using two different test groups. The groups included samples with a higher flute from the front (on the side of the DIC stereo set) and samples with a lower flute from the front. It is evident that the results for group 2, especially in the case of $A_{11}$ and $A_{12}$, differed significantly from the results obtained in the first procedure, while considering group 1. This was due to the asymmetric cross-section of the sample and the different level of buckling on the sample side with the higher flute. Out-of-plane deformation related to buckling distorts measurement and therefore introduces noise that distorts the results. Other components of matrix A did not differ more than 10% when using different measurement techniques, which was very promising.

**Figure 18:**
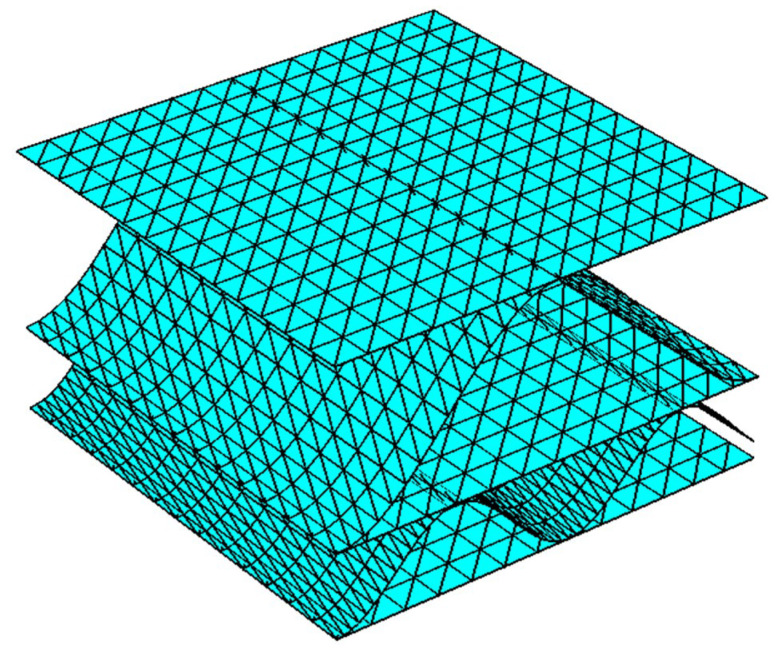
Visualization of the finite element model of corrugated board BE-650.

In order to validate the results presented in Table 4, the numerical homogenization procedure (for details see recent works by Garbowski and Gajewski [9] or Garbowski et al. [10]) of the cross-section of corrugated board BE-650 (see Figure 18) was used. The numerical homogenization technique used the geometrical and constitutive parameters presented in Table 1 and Table 2. The following results were obtained while employing the homogenization technique: $A_{11}=2620$ N/mm, $A_{12}=185$ N/mm, $A_{22}=1812$ N/mm, $A_{66}=906$ N/mm. The results are in good agreement, which proves that the use of optical techniques in conjunction with the new setup of the ECT (samples cut at an angle of 45° with respect to the direction of corrugation) can be effective in determining the stiffness of corrugated cardboard.

## 5. Conclusions

The main conclusion is that stereo DIC and/or optical extensometry techniques can be used to evaluate stiffness in a standard edge crush test. In order to determine all the stiffness coefficients, it is necessary to use an additional, new test specimen cut at an angle of 45° to the direction of the corrugation. By applying the results from the two samples simultaneously and using a least squares minimization approach, all of the stiffness components can be easily identified. The only concern is proper surface selection in unsymmetrical corrugated cardboard samples for stereo DIC measurement, especially in the 45° tests. However, this is easily remedied by using a larger sample set and averaging the results.

## Figures and Tables

**Table 1:** The geometrical features of both corrugated layers of EB-650.

Wave (Flute)	Pitch (mm)	Height (mm)	Take-Up Ratio (–)
E	3.50	1.18	1.242
B	6.48	2.5	1.315

**Table 2:** Mechanical properties of individual layers of 5EB650C3.

**Layer** **Name**	**Thickness** **(** μm **)**	$\begin{array}{c} E_{MD}\\ \left(\mathrm{kN}/m\right) \end{array}$	$\begin{array}{c} E_{CD}\\ \left(\mathrm{kN}/m\right) \end{array}$	$\begin{array}{c} SCT_{CD}\\ \left(\mathrm{kN}/m\right) \end{array}$
TLWC 140	180	725	323	2.32
W 100	160	886	328	1.76
TLW 120	170	907	313	1.81

**Table 3:** Elastic stiffness index in CD computed from the displacement measurement by the optical extensometry and from machine crosshead, as well as the edgewise compression strength in CD.

Test ID	E—Optical (N/mm)	E—Crosshead (N/mm)	ECT (N/mm)
1	1447.45	441.82	−7.548
2	1380.25	536.82	−7.151
4	1531.96	450.66	−7.609
5	1615.12	611.39	−7.640
Mean (N/mm)	1493.70	510.17	−7.487
Std (N/mm)	102.01	79.93	0.227
Cov (%)	6.829	15.668	−3.038

**Table 4:** The components of A matrix.

Parameter:	Test 2 and 6	Group 1	Group 2
$A_{11}$ (N/mm)	2581	2583.0	3554.0
$A_{12}$ (N/mm)	158	103.5	158.1
$A_{22}$ (N/mm)	1674 (1500 ^1^)	1765.0	1792.0
$A_{66}$ (N/mm)	1078	1061.0	946.0

^1^ Results obtained directly from test 2 in the CD using Equation (7) or (9).

## Data Availability

The data presented in this study are available on request from the corresponding author.
